# Karyotype reshufflings of *Festuca pratensis* × *Lolium perenne* hybrids

**DOI:** 10.1007/s00709-017-1161-5

**Published:** 2017-09-07

**Authors:** Joanna Majka, Zbigniew Zwierzykowski, Maciej Majka, Arkadiusz Kosmala

**Affiliations:** Institute of Plant Genetics, Polish Academy of Sciences, Strzeszyńska 34, 60-479 Poznań, Poland

**Keywords:** Genetic instability, Fragile sites, Interstitial telomeric sequences, *Festuca pratensis* × *Lolium perenne* hybrids, Karyotype reshuffling

## Abstract

Many different processes have an impact on the shape of plant karyotype. Recently, cytogenetic examination of *Lolium* species has revealed the occurrence of spontaneous fragile sites (FSs) associated with 35S rDNA regions. The FSs are defined as the chromosomal regions that are sensitive to forming gaps or breaks on chromosomes. The shape of karyotype can also be determined by interstitial telomeric sequences (ITSs), what was recognized for the first time in this paper in chromosomes of *Festuca pratensis* × *Lolium perenne* hybrids. Both FSs and ITSs can contribute to genome instabilities and chromosome rearrangements. To evaluate whether these cytogenetic phenomena have an impact on karyotype reshuffling observed in *Festuca* × *Lolium* hybrids, we examined F_1_ *F. pratensis* × *L. perenne* plants and generated F_2_-F_9_ progeny by fluorescent in situ hybridization (FISH) using rDNA sequences, telomere and centromere probes, as well as by genomic in situ hybridization (GISH). Analyses using a combination of FISH and GISH revealed that intergenomic rearrangements did not correspond to FSs but overlapped with ITSs for several analyzed genotypes. It suggests that internal telomeric repeats can affect the shape of *F. pratensis* × *L. perenne* karyotypes. However, other factors that are involved in rearrangements and have a more crucial impact could exist, but they are still unknown.

**Electronic supplementary material:**

The online version of this article (10.1007/s00709-017-1161-5) contains supplementary material, which is available to authorized users.

## Introduction

Cytogenetic analyses, including fluorescent in situ hybridization (FISH) and genomic in situ hybridization (GISH) methods, provide valuable contribution to resolve karyotype variation and evolution (Kopecký et al. 2006; Schubert 2007), as well as the genomic composition of hybrids (Zwierzykowski et al. 2006, 2008). FISH enables the determination of karyotype structure on the basis of the number and physical location of various sequences both repetitive and single locus (Xiong and Pires 2011). GISH with total genomic DNA as a probe can discriminate parental genomes in hybrids and can reveal the occurrence of intergenomic rearrangements (Kopecký et al. 2006). The combination of FISH and GISH techniques has been effectively used for detecting genome compositions and variation in various plant species (Dou et al. 2009; Kwiatek et al. 2016; Majka et al. 2016), including *Festuca* and *Lolium* hybrids (Kosmala et al. 2006; Książczyk et al. 2015).

Species of the *Festuca*-*Lolium* complex belong to the most important temperate fodder grasses and possess complementary and agriculturally desirable traits. *Festuca pratensis* and *Lolium perenne* are closely related and can hybridize at different ploidy levels, producing intergeneric hybrids. Despite the close relationship, the evolutionary distance between these genera is sufficient to distinguish parental genomes in amphiploid and introgression forms (Zwierzykowski et al. 1998; Kosmala et al. 2006). In hybrids of *Festuca* and *Lolium* species, interspecies rearrangements have been observed, which are the effects of genomes’ interactions (Zwierzykowski et al. 1998, 2011; Kopecký et al. 2006).

Chromosomal aberration which leads to karyotype reshuffling can be caused by different mechanisms/processes. It was reported that genome instability could be associated with interstitial telomere sequences (ITSs) or fragile sites (FSs). The ITSs sites can colocalize with breakages and chromosomal rearrangements (Bouffler et al. 1996; Lin and Yan 2008). The FSs are defined as the chromosomal regions that are sensitive to forming gaps or breaks on chromosomes. Fragile sites are thought to be caused by disruptions in DNA synthesis or could be related to gene activity decondensation dependent of the 35S rDNA containing chromatin within the NOR, what was reported by Rocha et al. (2017). They can occur spontaneously or can be induced by aphidicolin, which is an inhibitor of DNA polymerase α and other polymerases (Glover et al. 1984, 2005; Huang et al. 2012). FSs have been identified in human and other mammalian species, including dogs (Stone et al. 1991) and cats (Stone et al. 1993). *L. perenne* and *L. multiflorum* were the first plant species where FSs have been described (Huang et al. 2008). Recently, FSs were recognized in *Phleum echinatum* (Grabowska-Joachimiak et al. 2015), *Citrus sinensis* (Lan et al. 2016), and *Festuca arundinacea* (Rocha et al. 2017).

It was observed that in tumor cells, FSs are involved in chromosome rearrangements (Dillon et al. 2010). Chromosome reshufflings in the karyotype of plant species were also observed, e.g., *Phleum echinatum* (Grabowska-Joachimiak et al. 2015). For *Lolium* species, a link between FSs and 35S rDNA cluster was reported (Huang et al. 2012, Rocha et al. 2015).

The main aim of this work was to elucidate whether FSs of 35S rDNA are related with karyotype reshufflings of F_2_-F_9_ progeny of the tetraploid (2n = 4× = 28) *F. pratensis* × *L. perenne* hybrid and whether genomic instability occurring in this hybrid plants can be coincided with detected FSs of 35S rDNA or location of ITSs.

## Material and methods

### Plant material

Plant material consisted of allotetraploid intergeneric hybrids (2n = 4× = 28) obtained by crossing autotetraploid forms of *F. pratensis* (2n = 4× = 28) and *L. perenne* (2n = 4× = 28) under controlled conditions. Plants of the F_1_ generation were intercrossed to produce F_2_ progeny. This rule was in force for the production of next generations (F_3_-F_9_). Hybrid plants constitute the part of the collection, performed and maintained in a greenhouse at the Institute of Plant Genetics, Polish Academy of Sciences (IPG PAS). The genomic structure for all the plants from this collection was described by Zwierzykowski et al. (2006, 2011). From the collection of IPG PAS were selected hybrid plants, in which karyotype intergenomic rearrangements occurred. All the hybrid plants studied are listed in Table 1. The F_1_ generation, as well as diploid (2n = 2× = 14) and tetraploid (2n = 4× = 28) forms of *F. pratensis* and *L. perenne*, were used as controls.

**Table 1:** Cytogenetic analysis in plants of F_2_-F_9_ generations derived from *F. pratensis* (2n = 4× = 28) × *L. perenne* (2n = 4× = 28) hybrids

No. of plants studied	Origin	Chromosome numer (2n)	No. of *L. perenne* chromosomes	No. of *F. pratensis* chromosomes	No. of rearranged chromosomes	No. of recombined chromosome arms	No. of terminal rearrangements	No. of interstitial rearrangements	No. of pericentromeric rearrangements	No. of chromosomes with 35S rDNA
1	F_2_-A15	28	15	13	4	4	4	0	0	8
2	F_2_-A29	27	13	14	2	2	0	2	0	7
3	F_2_-B21	28	14	14	4	4	3	0	1	10
4	F_2_-B29	28	12	16	2	2	2	0	0	10
5	F_3_-9	27	15	12	9	10	9	1	0	8
6	F_3_-11	28	18	10	12	14	11	1	2	10
7	F_4_-3	27	16	11	11	13	11	0	2	9
8	F_4_-15	28	16	12	10	14	12	2	0	11
9	F_4_-16	28	15	13	12	13	12	0	1	10
10	F_4_-21	28	18	10	12	14	14	0	0	12
11	F_5_-23	28	14	14	10	11	10	1	0	8
12	F_5_-24	28	15	13	12	14	12	1	1	10
13	F_6_-1	26	18	8	12	13	11	2	0	10
14	F_6_-2	26	15	11	14	18	15	2	1	9
15	F_6_-8	27	15	12	15	20	19	1	0	10
16	F_7_-25	28	20	8	15	15	11	3	1	12
17	F_7_-31	29	15	14	17	19	17	2	0	10
18	F_7_-40	28	23	5	14	16	13	3	0	14
19	F_7_-47	28	22	6	16	18	15	1	2	14
20	F_8_-5	27	20	7	15	17	14	1	2	11
21	F_8_-9	28	16	12	14	14	12	0	2	11
22	F_8_-17	28	20	8	16	21	18	1	2	10
23	F_8_-21	28	18	10	16	17	16	1	0	11
24	F_8_-28	28	19	9	17	18	15	2	1	11
25	F_8_-29	28	19	9	15	18	16	2	0	9
26	F_8_-33	28	15	13	20	22	18	1	3	8
27	F_9_-3	28	18	10	18	25	21	4	0	9
28	F_9_-8	27	19	8	12	17	11	5	1	11
29	F_9_-9	27	18	9	18	25	18	7	0	11
30	F_9_-10	28	19	9	16	22	17	4	1	12
31	F_9_-17	28	19	9	21	27	23	3	1	10
32	F_9_-31	28	17	11	15	22	21	1	0	10
Total:		886	546	340	416	499	421	54	24	326

### Chromosome preparations

Root tips were collected from the water culture hydroponic system. Metaphase accumulation and fixation procedures were carried out according to Majka et al. (2017a). Root meristems were digested with a mixture of enzymes containing 20% (*v*/*v*) pectinase (Sigma), 1% (*w*/*v*) cellulose (Calbiochem), and 1% (*w*/*v*) cellulase “Onozuka R-10” (Serva) at 37 °C and squashed in a drop of 60% acetic acid. Slides of good quality were frozen in liquid nitrogen.

### Probes

Clone 395, derived from the library representing the most frequently present sequences in the *F. pratensis* genome, was labeled by nick translation with fluorochrome Atto647 (Jena BioScience) (Majka et al. 2017b). The ribosomal sequence 35S rDNA was labeled with digoxigenin-11-dUTP by nick translation. While 5S rDNA and the *Arabidopsis thaliana* L. telomere repeats (TTTAGGG)_n_ were labeled by polymerase chain reaction (PCR) with tetramethyl-rhodamine-5-dUTP (Sigma). The total genomic DNA of *L. perenne* was used as a probe for genomic in situ hybridization and was labeled with digoxigenin-11-dUTP using nick translation kit according to manufacturer instruction (Sigma).

### Fluorescent in situ hybridization

The FISH procedure was performed according to Hasterok et al. (2006) with minor modifications. The hybridization mixture consisting of 100–120 ng of each probe in the presence of salmon sperm DNA, 50% formamide, 2 × SSC, and 10% dextran sulfate was applied onto chromosome slides. Chromosome slides together with the hybridization mixture were denatured for 2 min at 80 °C and then incubated in a humid chamber at 37 °C overnight. The post-hybridization washes were performed in 0.1 × SSC buffer at 42 °C (73% stringency). Probes labeled with digoxigenin-11-dUTP were detected using antidigoxigenin fluorescein isothiocyanate (FITC) (Sigma). Chromosome slides were counterstained with DAPI in Vectashield (Vector Laboratories). After the acquisition of images, selected slides were washed off and reprobed with a new set of probes according to Heslop-Harrison (2000).

### Genomic in situ hybridization

GISH was performed according to Kosmala et al. (2006) with minor modifications. The nuclear DNA of *L. perenne* was used as a probe, while DNA of *F. pratensis* was used as blocking DNA. The DNA of *F. pratensis* was sheared to 200–500 bp fragments by boiling for 45 min. The hybridization mixture consisted of 50% deionized formamide, 10% dextran sulfate, 2 × SSC, 0.5% SDS, as well as 100–120 ng/slide gDNA probe. Chromosomal DNA was denatured in the presence of the hybridization mixture at 80 °C for 2 min and then allowed to hybridize at 37 °C overnight. Post-hybridization washes were performed at room temperature in 2 × SSC buffer. Chromosomes were counterstained with propidium iodide in Vectashield antifade solution (Vector Laboratories).

### Imaging

In the first round of hybridization, clone 395, derived from nuclear DNA of *F. pratensis*, was hybridized. In the next step, GISH was performed and in the last one hybridization 5S rDNA, 35S rDNA, and telomeric sequences were mapped. Examination of slides was carried out using an Olympus BX 61 automatic epifluorescence microscope with Olympus XM10 CCD camera. Digital images were imported into the Micrographx Picture Publisher software (version 10; Corel Corporation, Canada) and Microsoft Publisher for final processing.

## Results

Analysis of *F. pratensis* × *L. perenne* hybrids was focused on the examination of rearranged chromosomes. Among 32 hybrid plants, derived from F_2_-F_9_ generations, in which karyotype reshuffling processes took place, almost 47% (416 out of 886) of a total number of chromosomes were recombined (Table 1). Across hybrids of F_2_-F_9_ generations, disparate patterns of structural rearrangements were observed. For the analyzed plants, changes located in a terminal part of chromosomes prevailed (421 changes out of 499 recombined arms; 84%). Although rearrangements in interstitial (54 out of 499; 11%) and pericentromeric (24 out of 499; 5%) regions were also detected, they occurred more rarely. For F_1_ plants, 28 chromosomes were observed (14 *Festuca*-derived chromosomes and 14 *Lolium*-derived chromosomes). In karyotypes of these plants, none of the intergenomic rearrangements were noticed.

**Fig. 1:**
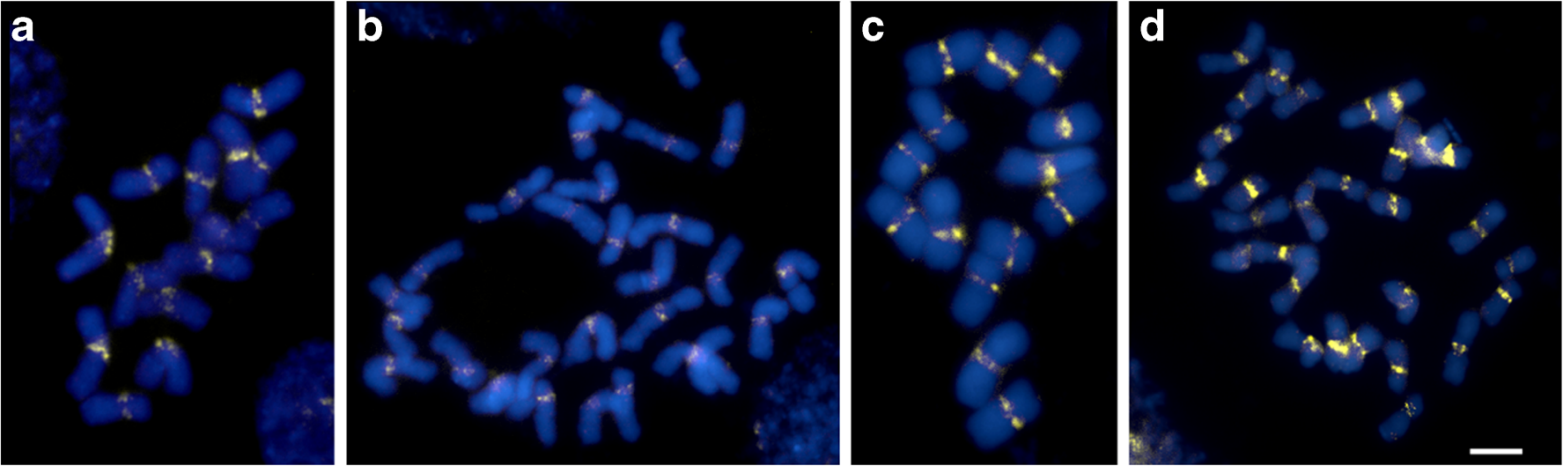
FISH mapping of clone 395 (*yellow*) to *F. pratensis* and *L. perenne* chromosomes. **a** Diploid (2n = 14) *F. pratensis*. **b** Tetraploid (2n = 28) *F. pratensis*. **c** Diploid (2n = 14) *L. perenne*. **d** Tetraploid (2n = 28) *L. perenne*. The clone was labeled with Atto647 fluorochrome; chromosomes were counterstained with DAPI (*blue*)

In FISH experiments *Arabidopsis*-type telomeric probe, 35S rDNA and 5S rDNA, as well as centromeric clone (395) were used. The physical location of clone 395, which was derived from the nuclear DNA library of *F. pratensis*, was determined in diploid and tetraploid forms of *F. pratensis* and *L. perenne*. This sequence was specific for centromeric regions of both species (Fig. 1).

**Fig. 2:**
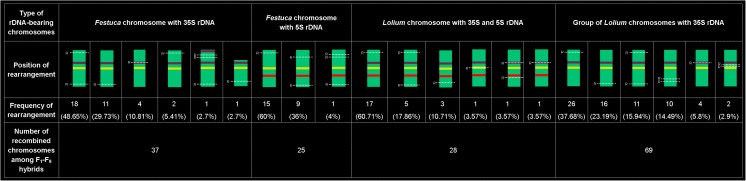
The types and frequency of rearrangements occurring in rDNA-bearing chromosomes for *F. pratensis* × *L. perenne* hybrids. *Yellow lines* show the position of centromeric regions; *purple lines* show the position of 35S rDNA; *red lines* show the position of 5S rDNA; *white dotted lines* show the position of rearrangements (R)

Analysis of rDNA-bearing chromosomes combined with GISH results allowed to determine the pattern and frequency of their changes. This analysis encompassed *Festuca*-derived chromosomes with 35S rDNA, *Festuca*-derived chromosomes with 5S rDNA, *Lolium*-derived chromosomes with 5S rDNA and 35S in opposite arms, and a group of *Lolium*-derived chromosomes bearing 35S rDNA loci. It was revealed that for *Festuca*-derived chromosomes bearing 5S rDNA, three types of changes existed (Fig. 2). For the other groups, six patterns of rearrangements occurred. Within each group, one type of changes prevailed. A cytogenetic examination of chromosomes with rDNA sequences revealed that the most recombined chromosomes were in the group of *Lolium*-derived chromosomes bearing 35S rDNA (69 rearranged chromosomes). It should be highlighted that this group consisted of three pairs of chromosomes, which cannot be recognized individually. A high number of rearrangements was also perceived for *Festuca* chromosomes with 35S rDNA (37 rearranged chromosomes). Additionally, it is worth mentioning that changes of 35S rDNA location in *Festuca*-derived chromosomes with this sequence were also observed (Fig. 2). In one genotype, *Festuca*-derived chromosome with two loci of 35S rDNA in one arm was identified (ESM 1). In another one plant, *Festuca*-derived chromosome was observed, in which probable deletion in the chromosome arm bearing 35S rDNA took place, what was determined on the basis of chromosome morphology (Online Resource 1b).

**Fig. 3:**
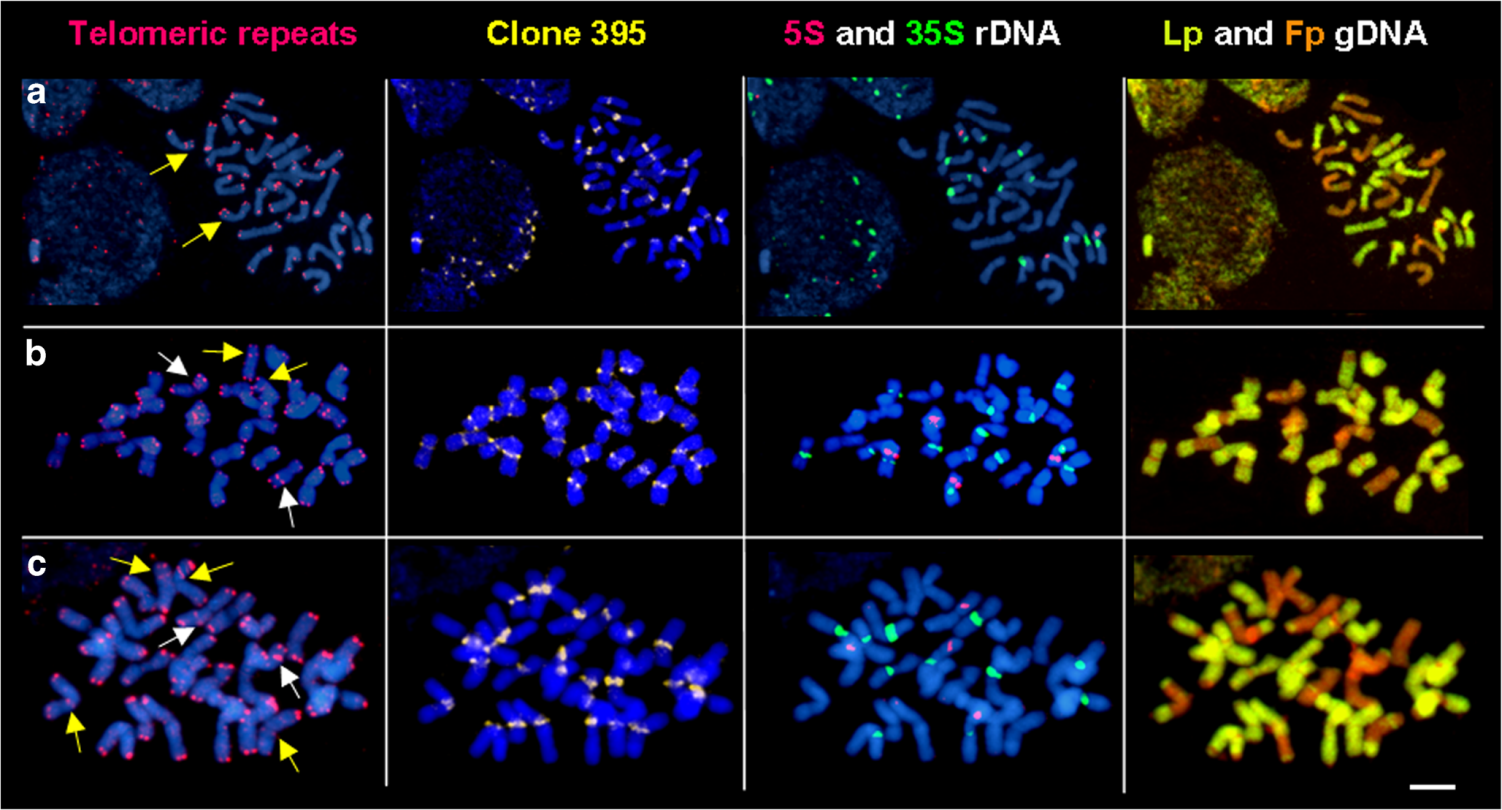
The interstitial telomeric sequences in *F. pratensis* × *L. perenne* hybrids: **a** Genotype F_4_-15—lack of colocalization of ITSs (*yellow arrows*) with centromeric sequences, rDNA loci, and GISH. **b** Genotype F_7_-40—colocalization of ITSs (*yellow arrows*) with location of rearrangements in *L. perenne* chromosomes and two additional ITSs (*white arrows*) in *F. pratensis* chromosomes. **c** Genotype F_8_-21—colocalization of ITSs (*yellow arrows*) with centromeric and pericentromeric regions in *F. pratensis* chromosomes and two additional ITSs (*white arrows*) in interstitial regions of *F. pratensis* chromosomes. In each panel, from the left to right side: telomeric repeats (*red*), centromeric sequence (*yellow*), 5S and 35S rDNA sequences (*red* and *green*, respectively), and GISH results were presented

The hybridization with a telomeric probe resulted in signals located distally in all the chromosomes of analyzed hybrids, but what is more important, in almost half of analyzed plants among all generations, interstitial signals were also recognized. The location of interstitial telomeric signals was compared to the results obtained for the rest of the probes, including centromeric sequences and rDNA, as well as the nuclear DNA of *L. perenne*. In Fig. 3a were presented the results obtained for one of the analyzed genotypes (F_4_-15), in which two interstitial telomeric signals (subtelomeric positions) were detected, but their locations did not correspond to the centromeric sequences, 5S and 35S rDNA sites, as well as did not overlap with rearrangements revealed by GISH. Additional telomeric sequences were located in *Festuca*-derived chromosomes. Hybridization with a telomeric probe allowed to recognize plants among *F. pratensis* × *L. perenne* hybrids, in which the higher number of ITSs occurred (Fig. 3b, c). In Fig. 3b, the plant (F_7_-40) with four interstitial telomeric sequences was shown (interstitial/subtelomeric position). Two signals were detected in *F. pratensis*-derived chromosomes and their location did not correspond to all of the mapping probes. The other two signals were mapped in *Lolium*-derived chromosomes. Furthermore, these chromosomes were recombined and the location of rearrangements was detected in the same places as interstitial telomeric signals. Only in one plant (F_8_-21) out of 32 analyzed plants, it was observed that the position of the interstitial telomeric signal was the same as the centromeric and pericentromeric region in *Festuca* chromosome (Fig. 3c).

**Fig. 4:**
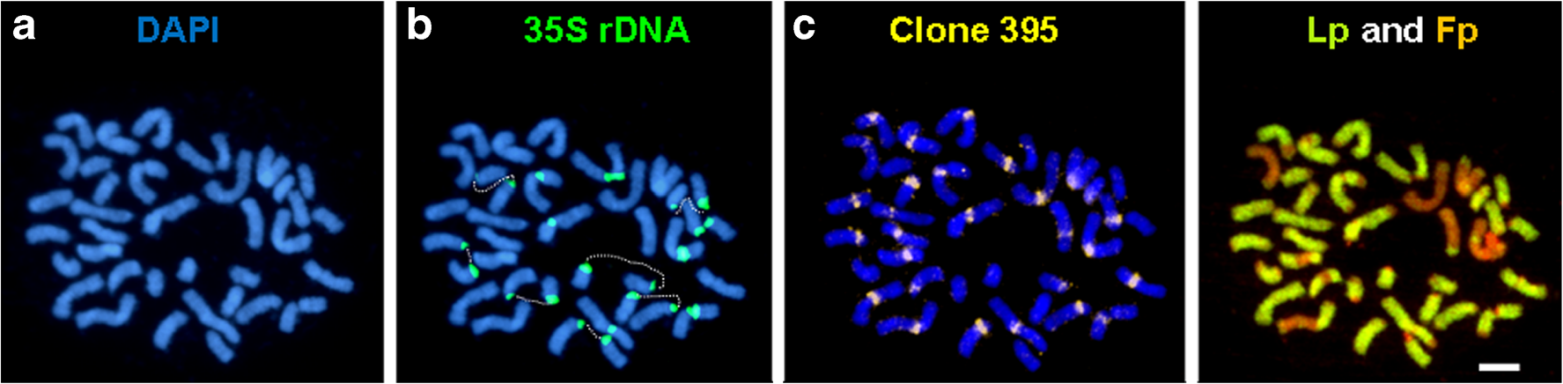
Metaphase of *F. pratensis* × *L. perenne* hybrid plant (F_7_-47) with fragile sites associated with 35S rDNA: **a** DAPI staining. **b** 35S rDNA-FISH (*green*) (the *white dotted lines* link broken chromosome parts). **c** Centromeric clone 395. **d** GISH

Moreover, in some metaphase plates of studied hybrids, the observed fragile sites resulted in breaks. For all hybrids, where FSs were recognized, they always overlapped with 35S rDNA sites, but in almost all cases did not correspond with places of intergenomic rearrangements (Fig. 4).

## Discussion

For *Festuca* × *Lolium* hybrids, it was previously presented that chromosomes can freely pair and recombine, what is crucial for the production of intergeneric hybrids combining profitable agronomic traits (Zwierzykowski et al. 2006, 2011). However, the occurrence of these processes has also an impact on genome instability of hybrids (Kopecký et al. 2006). In our analysis, we focused on the F_2_-F_9_ progeny of the *F. pratensis* × *L. perenne* hybrid and the main aim was to analyze that cytogenetic phenomena, like FSs and ITSs, have an influence on karyotype reshufflings of *F. pratensis* × *L. perenne* hybrids.

In this paper, we described for the first time ITSs for *F. pratensis* × *L. perenne* hybrids. The hybridization with various cytogenetic probes revealed that they were located nearby centromeric/pericentromeric or subtelomeric regions. A similar position of discovered ITSs was published for other plant species, e.g., *A. thaliana* (Richards et al. 1991), and *Solanum* species (He et al. 2013). It is worth mentioning that ITSs are genomic regions that are prone to instability, and may constitute hotspots for breakage, recombination, and rearrangement processes (Bolzan 2012; Dvorackova et al. 2015). The molecular mechanism of genome instability mediated by ITSs is not well understood (Aksenova et al. 2013). We suggest that for synthetic *F. pratensis* × *L. perenne* hybrids, ITSs may have arisen as a result of repairing the DNA breaks (chromosome healing) or microsatellite expansion (replication slippage resulted in insertion). Nonetheless, detected ITSs in hybrid genotypes examined with the GISH method, which determines the position of intergenomic rearrangements, revealed that only for a few plants the sites of ITSs corresponded to the location of rearrangements. For the majority of them, they did not colocalize with structural changes in chromosomes. It suggests that detected ITSs can be responsible for karyotype changes but the role they play seems to be nonessential. The presence of internal telomeric repeats on chromosomes can be also related to a telomere-telomere fusion of chromosomes, but this mechanism is more proper for naturally evolving species (Kilburn et al. 2001). It was reported that during the evolution of grasses, chromosome fusions took place (International Brachypodium Initiative 2010). In our analysis of diploid and tetraploid *F. pratensis* and *L. perenne* species, interstitial telomeric sequences were not detected (data not shown).

Another genomic regions that have a tendency to cause breaks and may lead to structural chromosome changes are known as fragile sites. Among plant species, FSs have been described firstly for *Lolium* species, and it was reported that 35S rDNA clusters are fragile sites (Huang et al. 2008, 2009, 2012). In our study, in karyotypes of *F. pratensis* × *L. perenne* hybrids, three groups of chromosomes were crucial for the examination of FSs connected with rDNA sequences—*Festuca* chromosomes bearing 35S rDNA, *Lolium* chromosomes bearing 5S and 35S rDNA, and the group of *Lolium* chromosomes with 35S rDNA. In the analyzed hybrids, we observed breaks of chromosomes in a location corresponding to 35S rDNA sites. Additionally, detailed analysis of rDNA-bearing chromosomes derived from both parental species resulted in an observation that only *Lolium*-derived chromosomes had a tendency to breaks. However, Zwierzykowski et al. (2006) reported that in the F_2_-F_6_ progeny of the *F. pratensis* × *L. perenne* hybrid, chromosomes of *Festuca* were more frequently recombined. Książczyk et al. (2015) on the basis of the analysis of F_2_-F_4_ generations of the *F. pratensis* × *L. perenne* hybrid suggested an asymmetrical variation of parental genomes and higher predisposition of *Festuca* chromosome to structural rearrangements. Despite a low number of plants in each generation, we also observed that the chromosomes of *F. pratensis* were more recombined, although their number was decreasing in successive generations.

Among both *Festuca*- and *Lolium*-derived chromosomes, rearrangements in the distal part of chromosomes were in dominance; however, interstitial and pericentromeric changes were also recognized. It is known that the recombination rate is not at the same level along the chromosome arms. Zwierzykowski et al. (1999) presented that the lowest frequency of the translocation breakpoints was around the centromere and telomeres. Additionally, Kopecký et al. (2010) showed the distribution of recombination events along individual chromosomes of *L. multiflorum*/*F. pratensis* introgression forms. The high rate of recombination in interstitial regions was observed, but not in every chromosome pair. In selected chromosomes, the highest level of recombination near telomeric/subtelomeric regions was recognized (Kopecký et al. 2010). King et al. (2013) showed that in genomes of *L. perenne*/*F. pratensis* introgression forms, the large proportion of genes was distributed in the proximal regions of chromosomes, where low or even very low frequencies of recombination were observed. Taking into account the analyzed hybrid plants, it seems that dominant terminal position of changes is in agreement with King et al. (2013).

Numerous studies of human tumor cells have shown that FSs can be connected with frequent breakage and rearrangements, e.g., FRA16D harboring the WWOX gene (Arlt et al. 2006; Hosseini et al. 2013). Whereas, our results revealed that breaks in 35S rDNA sites did not correspond to the position of intergenomic rearrangements, although it is suggested that fragile sites are preferred as the sites of recombination. Similarly, Rocha et al. (2017) reported that 35S rDNA sites were not the cause of karyotype instability for the chosen *Lolium* and *Festuca* species. In addition, Rocha et al. (2016) reported that FSs of 35S rDNA in *L. multiflorum* were not hotspots for chromosomal breakages, which were induced by X-ray irradiation.

Another important aspect, which was noticed among analyzed *F. pratensis* × *L. perenne* hybrids, was variability of 35S rDNA, e.g., on *F. pratensis* chromosome two loci of 35S rDNA were located in the same chromosome arm. What is more, this change was located only in one chromosome from the same pair. It can be suggested that identified variability may be caused by fragile sites associated with 35S rDNA loci. However, it was reported that the jumping of rDNA sequences, especially 35S rDNA, can also be activated and mediated by transposons in *Aegilops speltoides* (Raskina et al. 2004).

The results presented in this paper demonstrated clearly that the reshuffling of *F. pratensis* × *L. perenne* hybrids is not related to fragile sites connected with 35S rDNA sites. It suggests that other factors, like interstitial telomeric sequences, may be involved in chromosome rearrangements. Thus, more detailed examinations of processes which shape hybrid karyotypes are required.

## Electronic supplementary material

ESM 1 Structural changes in *Festuca*-derived chromosomes bearing 35S rDNA sequence: a) two loci of 35S rDNA in the same chromosome arm (white arrow); b) deletion in arm bearing 35S rDNA (white arrow). In the left pictures: 35S rDNA (green), 5S rDNA (red), chromosome were counterstained with DAPI (blue). In the right pictures: genomic DNA of *L. perenne* (green), chromosomes of *F. pratensis* with propidium iodide (orange) (JPEG 59 kb).

High Resolution Image (TIFF 1491 kb).
