# Supplementary figures and images for: Karyotype reshufflings of Festuca pratensis × Lolium perenne hybrids
Source: Protoplasma. 2017 Sep 7;255(2):451–8. doi: 10.1007/s00709-017-1161-5 (PMC5830480; doi:10.1007/s00709-017-1161-5)

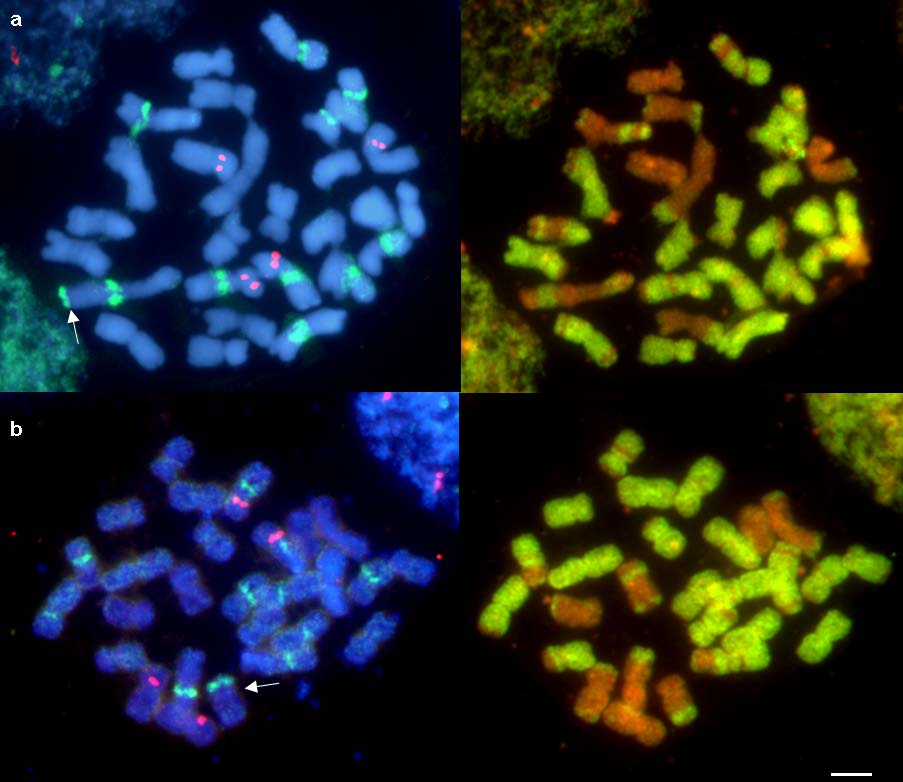

Supplement: Supplementary file 1 — Structural changes in Festuca-derived chromosomes bearing 35S rDNA sequence: a) two loci of 35S rDNA in the same chromosome arm (white arrow); b) deletion in arm bearing 35S rDNA (white arrow). In the left pictures: 35S rDNA (green), 5S rDNA (red), chromosome were counterstained with DAPI (blue). In the right pictures: genomic DNA of L. perenne (green), chromosomes of F. pratensis with propidium iodide (orange) (JPEG 59 kb). [file 709_2017_1161_Fig5_ESM.jpg]

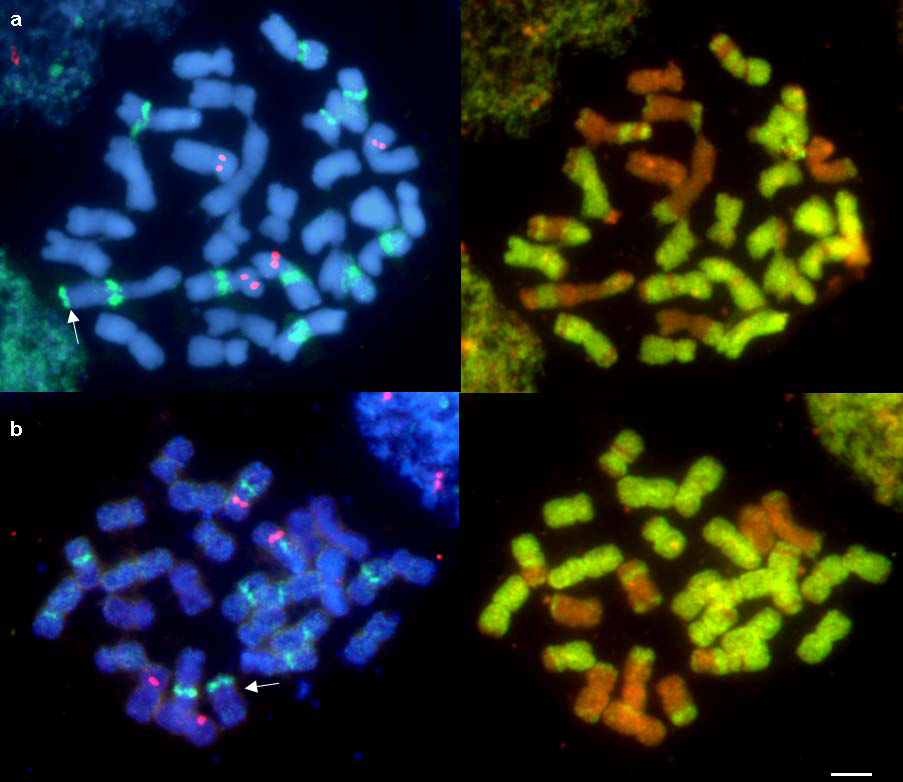

Supplement: Supplementary file 2 — High Resolution Image (TIFF 1491 kb). [file 709_2017_1161_MOESM1_ESM.tif]
